# Combination of leflunomide and benazepril reduces renal injury of diabetic nephropathy rats and inhibits high-glucose induced cell apoptosis through regulation of NF-κB, TGF-β and TRPC6

**DOI:** 10.1080/0886022X.2019.1665547

**Published:** 2019-09-25

**Authors:** Huili Li, Yuanyuan Wang, Zhangqing Zhou, Fang Tian, Huanhuan Yang, Juzhen Yan

**Affiliations:** Department of Nephrology, Xixi Hospital of Hangzhou (Hangzhou XIXI Affiliated Hospital of Zhejiang Chinese Medical University), Hangzhou, Zhejiang Province, People’s Republic of China

**Keywords:** Leflunomide, benazepril, diabetic nephropathy, NF-κB, TGF-β, TRPC6

## Abstract

**Objective:** To investigate effects of combination use of leflunomide and benazepril on diabetic nephropathy (DN) both *in vivo* and *in vitro*.

**Methods:** The streptozotocin (STZ) induced Sprague-Dawley rats were treated with leflunomide (15 mg/kg/d), benazepril (15 mg/kg/d) or both the two drugs. Fasting blood glucose (FBG) and renal function indexes including blood urea nitrogen (BUN), serum creatinine (Scr), and proteinuria and kidney/body weight ratio (KW/BW) were measured. HE staining was used for histological analysis. The rat glomerular mesangial cells (RMCs) were treated with high-glucose (150 mg/ml) and the leflunomide and benazepril with both concentrations of 50 μmol/l were used to treat the high-glucose induced cells. TUNEL assay was used for measurement of cell apoptosis. Western blotting was conducted to determine expression of nuclear factor Kappa B (NF-κB), transforming growth factor-β (TGF-β) and transient receptor potential canonical 6 (TRPC6).

**Results:** The body weight was significantly lower and all indexes of FBG, BUN, Scr, proteinuria and KW/BW ratio, GFR, as well as inflammatory factors TNF-α and IL-6 were significantly increased in the DN group after STZ treatment for 4 weeks. The treatment with leflunomide, benazepril or the both dramatically reduced the above effects induced by STZ, and the alteration was the most significant in the combination group. Treatment of leflunomide and benazepril significantly reduced expression levels of NF-κB, TGF-β and TRPC6 in renal tissues of DN rats as well as in high-glucose induced RMCs. It was also observed leflunomide and benazepril reduced high-glucose induced cell apoptosis of RMCs.

**Conclusion:** The combination use of leflunomide and benazepril could improve the renal function and reduce the renal injury of DN rats and could reduce the levels of NF-κb, TGF-β and TRPC6 in both DN rats and high-glucose induced RMCs.

## Introduction

The diabetes mellitus, one of the most prevalent chronic metabolic diseases worldwide, has affected more than 382 million people worldwide with a still increasing incidence [1,2]. Diabetes has many complications such as cardiovascular diseases [3], obesity [4], diabetic retinopathy [5] and diabetic nephropathy (DN) [6]. Currently the main treatment method for DN is still medication [7].

Both leflunomide and benazepril are widely used drugs for many diseases such as rheumatoid arthritis [8] and hypertension [9]. Studies showed that leflunomide had protective effects on renal damage of DN rats [10]. Meanwhile, benazepril is also considered to be effective in improving DN and decreasing proteinuria [11]. However, despite these studies, few researches focused on the combination use of leflunomide and benazepril in treatment of DN and the possible mechanisms.

In the present study, we aimed to investigate effects of combination use of leflunomide and benazepril on DN and high-glucose induced RCMs. We demonstrated that the combination use of leflunomide and benazepril could improve the renal function and reduce the renal injury of DN rats through reducing the levels of NF-κb, TGF-β and TRPC6 and inhibiting cell apoptosis of high-glucose induced glomerular mesangial cells. This research could provide research basis for application of leflunomide and benazepril in treatment of DN, as well as give some new research target for DN development.

## Materials and methods

### Animals and treatment

In the present study, 30 male Sprague-Dawley rats were obtained from the Animal center of Xixi Hospital of Hangzhou. The rats were kept in a light-controlled room under a 12 h/12 h light/dark cycle and controlled temperature (23–25 °C). All animals were housed in micro-isolator cages with free access to food and water. In particular, any effort was put to avoid unnecessary pain of the animals. The whole study was approved by the Institutional Animal Care Committee at Xixi Hospital of Hangzhou. All rats were 7–8 weeks, of whose weights were 200–230 g.

The intraperitoneal injection of streptozotocin (STZ, Sigma-Aldrich, St. Louis, MO, USA) with a single dose of 60 mg/kg was used to establish the DN model [12]. For the control rats, citric acid buffer with the same dose was intraperitoneally injected. After 72 h of STZ injection, the fasting blood glucose (FBG) of the rats were determined and FBG values >16.7 mmol/l were considered successful in diabetic model establishment. All animals were further divided into four groups: (1) the leflunomide group with injection of STZ and intragastric administration of leflunomide (15 mg/kg/d, Sigma-Aldrich) for 12 weeks; (2) the benazepril group with injection of STZ and intragastric administration of benazepril (15 mg/kg/d, Sigma-Aldrich) for 12 weeks; (3) the combined group with injection of STZ and intragastric administration of both leflunomide (15 mg/kg/d) and benazepril (15 mg/kg/d) for 12 weeks, (4) the DN group with injection of STZ and intragastric administration of the same volume of normal saline, (5) the control group with injection of citric acid buffer and intragastric administration of the same volume of normal saline. All animals were sacrificed 12 weeks after treatment. The doses of benazepril and leflunomide were determined according to previous researches as well as our own experience [13].

### Renal function indexes and serum TNF-α and IL-6 levels

Renal function indexes including FBG, blood urea nitrogen (BUN), serum creatinine (Scr), proteinuria and kidney/body weight ratio (KW/BW) were measured. BUN and Scr levels were determined using an automatic biochemistry analyzer (Hitachi, Tokyo, Japan). BG, proteinuria and Scr concentrations, as well as serum levels of TNF-α and IL-6 were measured using commercial enzyme-linked immunosorbent assay (ELISA) kits (Abcam, Cambridge, MA, USA) according to the manufacturer’s instructions. For measurement of glomerular filtration rate (GFR), the 24 h urine volume (Vu) of the rats was recorded and urine creatinine levels were evaluated using the automatic biochemistry analyzer (Hitachi). GFR was calculated as: GFR = (urine creatinine/Scr) * Vu/body weight [14]. All the above measurement was conducted 12 weeks after treatment. Body weights were measured every day after STZ induction.

### Histological analysis

The renal injury was determined after 12 weeks for treatment by HE staining. Briefly, renal sections were deparaffinized and dehydrated. Then sections were stained in Harris hematoxylin solution for 8 min and were put into 0.2% ammonia water for 1 min. The sections were counterstained in eosin-phloxine solution for 1 min after rinsing in 95% alcohol. The photographs were taken under a Nikon microscope (ECLIPSE 9 0 i, Nikon, Melville, NY, USA).

### Cell culture and treatment

The rat glomerular mesangial cells (RMCs) were obtained from ATCC (Manassas, VA, USA). Cells were cultured in RPMI-1640 (Thermo Fisher Scientific, Inc., Waltham, MA, USA) supplemented with 10% Gibco^®^ fetal bovine serum (FBS) and 100 μg/mL penicillin–streptomycin (Sigma-Aldrich) at 37 °C and 5% CO_2_. The cells were then divided into three groups, (1) the control group treated with 5 mg/ml d-glucose (Sigma-Aldrich), (2) the high-glucose group treated with 150 mg/ml d-glucose for 48 h, (3) the leflunomide and benazepril group treated with 150 mg/ml d-glucose as well as leflunomide and benazepril with both concentrations of 50 μmol/L for 48 h.

### TUNEL assay

For TUNEL assay, cells were stained with an Apoptosis In Situ Detection Kit (Abcam, Cambridge, MA, USA) according to the manufacturer’s instructions. Briefly, after, paraffin embedded, deparaffinization and washed in PBS, the samples were treated with proteinase K, and incubated with TUNEL reaction mixture at 37 °C for 1 h. The number of TUNEL positive cells was calculated as percent of total number of cells. A Leica TCS-SP laser scanning confocal microscope (Leica Microsystems, Heidelberg, Germany) was used to take the photomicrographs.

### Western blotting

Western blotting was used to determine the protein levels of NF-κB, TGF-β and TRPC6. GAPDH was used as a control. Briefly, the proteins were extracted from the renal tissues or RMCs using radio-immunoprecipitation assay (RIPA) buffer (Vazyme Biotec Co., LTD, Nanjing, China) and were quantitated with protein assay reagent from Bio-Rad (Hercules, CA, USA). Then samples (30 μg) were subjected to 10% SDS-PAGE, transferred to polyvinylidene difluoride membranes, followed by blocking with 5% nonfat milk at room temperature for 1 h. The primary antibodies (all purchased from Abcam) were then incubated at 4 °C overnight, following with incubation of corresponding secondary antibodies (Abcam) at 37 °C for 45 min. Films were scanned using Super Signal West Pico Chemiluminescent Substrate kit (Pierce; Thermo Fisher Scientific, Inc., Waltham, MA, USA) according to the manufacturer’s protocol and were quantified using Image-Pro Plus software (version 6.0; Media Cybernetics, Inc., Rockville, MD, USA).

### Statistical analysis

The measurement data was expressed by mean ± SD. Comparison among three or more groups was conducted using one-way analysis of variance (ANOVA) followed by Tukey *post hoc* test. It was considered to be statistically significant when *p* values was <.05. All calculations were made using SPSS 18.0.

## Results

### Treatment of leflunomide and benazepril improved renal function of DN rats

**Figure 1.**
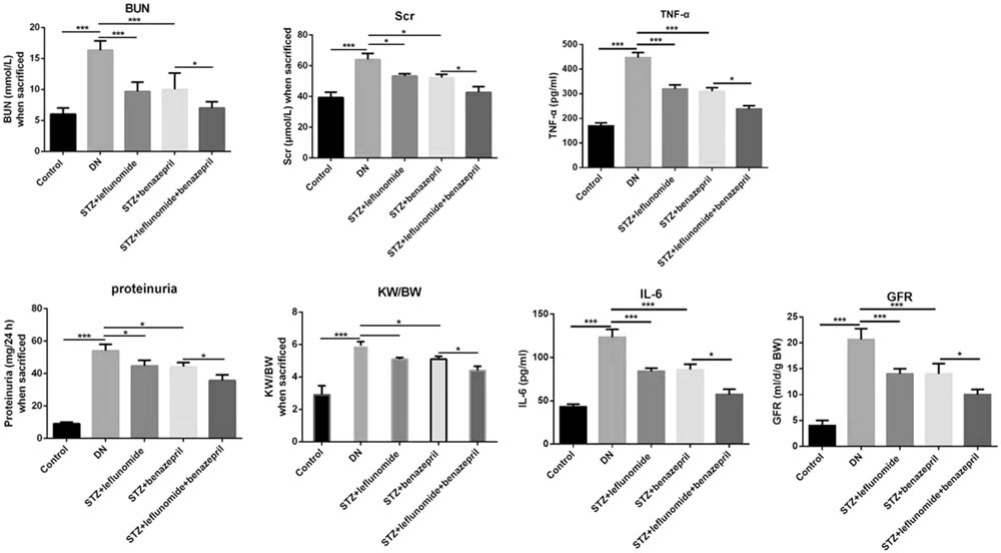
Renal function indexes, BUN, Scr, proteinuria, KW/BW, TNF-α and IL-6 for all groups of rats. In DN rats, all indexes of BUN, Scr, proteinuria and KW/BW, GFR, as well as inflammatory factors TNF-α and IL-6 were significantly increased after treatment of STZ, however treatment with leflunomide, benazepril or the both dramatically reduced the effects. Renal function indexes were significantly lower in the combination group.

To investigate effect of combination use of leflunomide and benazepril on DN rats, the renal function indexes including BG, BUN, Scr and proteinuria, as well as the body weight and KW/BW ratio were measured. As shown in Table 1, the body weight after 4 weeks treatment was significantly lower in the DN group than the control (*p* < .05). However, when treated with leflunomide, benazepril or both the two drugs, the body weight was dramatically enhanced compared with the DN group (*p* < .05), and the effect was the most significant in the combination group (*p* < .05). On the contrary, in DN rats, all indexes of BG, BUN, Scr, proteinuria and KW/BW, GFR, as well as inflammatory factors TNF-α and IL-6 were significantly increased after treatment of STZ, however treatment with leflunomide, benazepril or the both dramatically reduced the effects (Table 2 and Figure 1, p < .05). Meanwhile, all above renal function indexes were significantly lower in the combination group compared with the leflunomide or benazepril group (*p* < .05). All these results suggested treatment of leflunomide and benazepril could improve the renal function of the DN rats.

**Table 1.** Change of body weight for different groups of rats (g).

Group	0 week	4 weeks	8 weeks	12 weeks
Control	212.8 ± 10.5	332.7 ± 12.7	438.0 ± 8.4	514.7 ± 7.2
DN	212.0 ± 7.1	224.3 ± 11.4^a^	242.2 ± 9.0^a^	261.5 ± 3.8^a^
DN + leflunomide	215.3 ± 9.7	256.0 ± 7.2^a^^,^^b^	271.5 ± 5.4^a^^,^^b^	297.3 ± 13.1^a^^,^^b^
DN + benazepril	222.7 ± 6.2	255.2 ± 14.0^a^^,^^b^	275.3 ± 10.3^a^^,^^b^	300.2 ± 15.1^a^^,^^b^
DN + leflunomide and benazepril	217.3 ± 6.3	272.0 ± 9.0^a^^,^^b^^,^^c^^,^^d^	304.2 ± 5.6^a^^,^^b^^,^^c^^,^^d^	334.3 ± 13.0^a^^,^^b^^,^^c^^,^^d^

a *p* < .05, compared with the control group.

b *P* < .05, compared with the DN group.

c *p* < .05, compared with the DN + leflunomide group.

d *p* < .05, compared with the DN + benazepril group. 0 week means the time for successful establishment of the DN model.

**Table 2.** Change of FBG for different groups of rats (mmol/l).

Group	0 week	4 weeks	8 weeks	12 weeks
Control	5.2 ± 0.7	4.9 ± 0.6	4.8 ± 0.6	5.2 ± 0.6
DN	25.5 ± 1.4^a^	25.3 ± 1.4^a^	25.3 ± 2.0^a^	26.2 ± 1.5^a^
DN + leflunomide	24.8 ± 1.4^a^	24.3 ± 1.4^a^	23.7 ± 0.8^a^	22.7 ± 0.8^a^
DN + benazepril	26.3 ± 1.6^a^	23.7 ± 0.8^a^	24.0 ± 0.6^a^	23.0 ± 0.9^a^
DN + leflunomide and benazepril	26.5 ± 2.3^a^	22.7 ± 1.5^a^^,^^b^	22.0 ± 1.4^a^^,^^b^	21.7 ± 1.0^a^^,^^b^

a *p* < .05, compared with the control group.

b *p* < .05, compared with the DN group. 0 week means the time for successful establishment of the DN model.

### Treatment of leflunomide and benazepril reduced renal injury of DN rats

**Figure 2.**
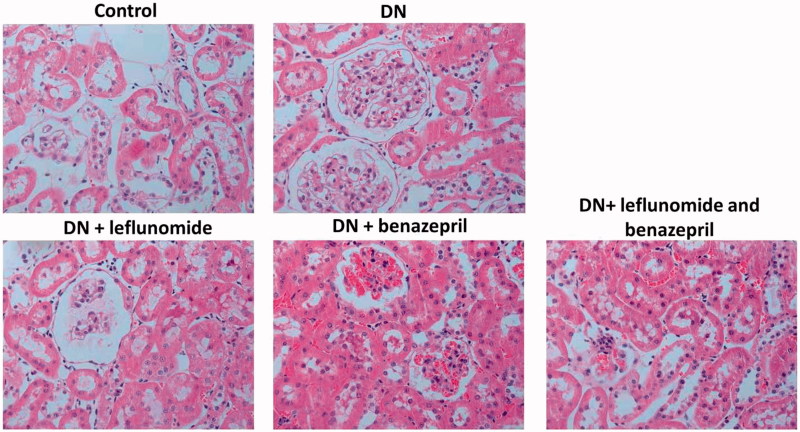
Histological analysis for all groups of rats using HE staining. In DN group, the glomerular basement membrane thickening, segmental differentiation and hypertrophic glomeruli were obviously observed. And in all treatment groups the above pathological changes were apparently alleviated.

To further investigate the effects of leflunomide and benazepril on DN rats, HE staining was conducted to see the renal injury. Results showed in DN rats, glomerular basement membrane thickening, segmental differentiation and hypertrophic glomeruli were obviously observed, indicating the renal injury of DN rats (Figure 2). However, when treated with leflunomide or benazepril the renal injury was apparently reduced, and the treatment of both leflunomide and benazepril resulted in the most obvious effects. These results further indicated the treatment of leflunomide and benazepril could reduce renal injury of the DN rats

### Treatment of leflunomide and benazepril reduced expression levels of NF-κB, TGF-β and TRPC6 in renal tissues of DN rats

**Figure 3.**
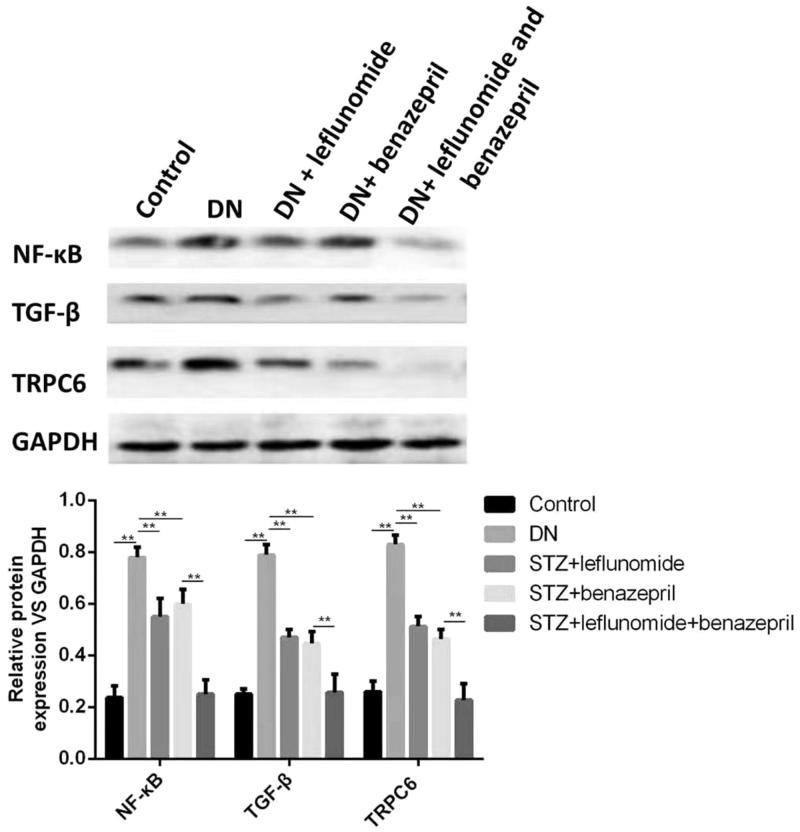
Protein expression of NF-κB, TGF-β and TRPC6 in renal tissues of different groups of rats and the quantified results. Protein levels of NF-κB, TGF-β and TRPC6 significantly increased in DN rats. The treatment of leflunomide, benazepril or both the two drugs could significantly reduce the protein levels, and the protein levels were significantly lower in the combination group.

The effects of treatment of leflunomide and benazepril on expression level of NF-κB, TGF-β and TRPC6 were further studied by western blotting. As shown in Figure 3, all protein levels of NF-κB, TGF-β and TRPC6 significantly increased in DN rats compared with the control rats (*p* < .05). The treatment of leflunomide, benazepril or both the two drugs could significantly reduce the protein levels (*p* < .05). Meanwhile the protein levels were significantly lower in the combination group than the groups treated with only leflunomide or benazepril (*p* < .05), suggesting the combination use of leflunomide and benazepril could reduce the protein levels of NF-κB, TGF-β and TRPC6.

### Treatment of leflunomide and benazepril reduced expression levels of NF-κB, TGF-β and TRPC6 in high-glucose induced RMCs

**Figure 4.**
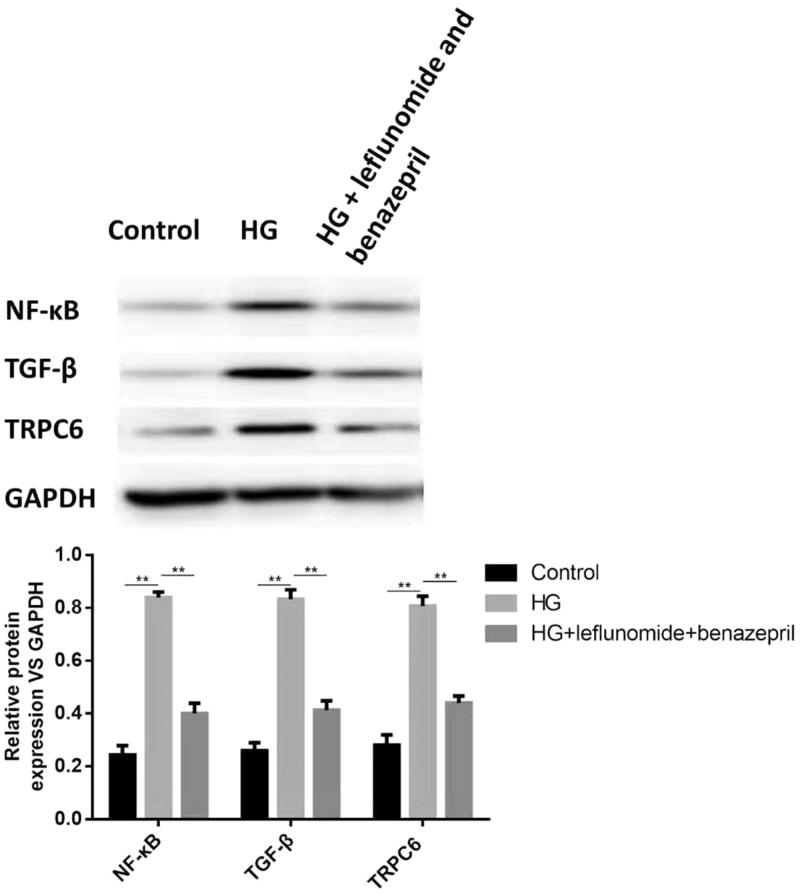
Protein expression of NF-κB, TGF-β and TRPC6 in different groups of RMCs and the quantified results. The expression levels of NF-κB, TGF-β and TRPC6 were significantly increased when cells were treated with high glucose. When treated with leflunomide and benazepril, the protein levels NF-κB, TGF-β and TRPC6 were dramatically decreased.

Then we conducted an *in vitro* study to show the effects of leflunomide and benazepril on expression levels of NF-κB, TGF-β and TRPC6 in high-glucose induced RMCs. Results showed the expression levels of NF-κB, TGF-β and TRPC6 were significantly increased when cells were treated with high glucose (Figure 4, *p* < .05). However, when treated with leflunomide and benazepril, the protein levels NF-κB, TGF-β and TRPC6 were dramatically decreased compared with the high glucose group (*p* < .05), suggesting leflunomide and benazepril could decrease the levels of NF-κB, TGF-β and TRPC6.

### Treatment of leflunomide and benazepril reduced high-glucose induced cell apoptosis of RMCs

**Figure 5.**
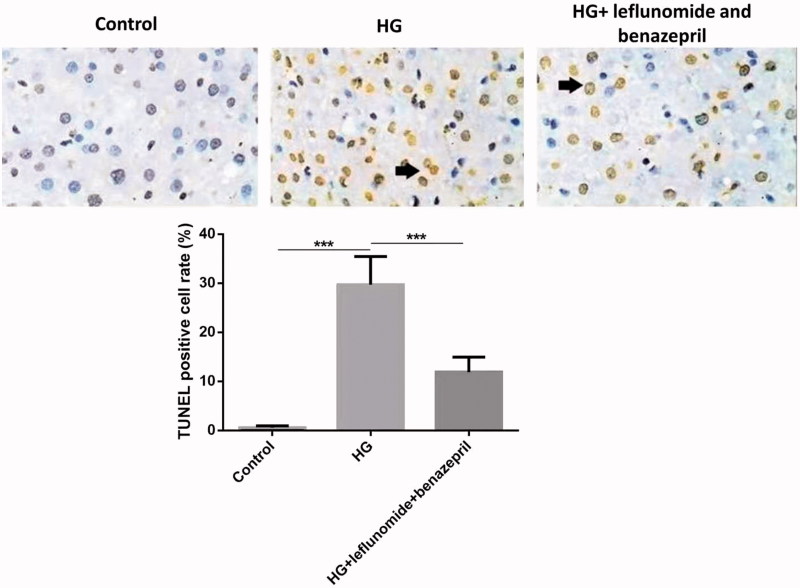
TUNEL assay of different groups of RMCs. Treatment of high glucose significantly increased the TUNEL positive cell rates compared with the control cells, while treatment of leflunomide and benazepril significantly reduced the TUNEL positive cell rates induced by high glucose.

At last we investigated effect of leflunomide and benazepril on high-glucose induced cell apoptosis of RMCs. As shown in Figure 5, treatment of high glucose significantly increased the TUNEL positive cell rates compared with the control cells (*p* < .05). However, treatment of leflunomide and benazepril significantly reduced the TUNEL positive cell rates induced by high glucose (*p* < .05). This result indicated treatment of leflunomide and benazepril could reduce high-glucose induced cell apoptosis of RMCs.

## Discussion

Despite numerous studies on DN, the mechanism for DN is still unclear and more effective treatment methods are still needed. Both leflunomide and benazepril were widely used drugs in treatment of many diseases. However, few studies focused on combination use of leflunomide and benazepril in treatment of DN. In the present study, we demonstrated that combination use of leflunomide and benazepril could improve the renal function and reduce the renal injury, as well as reduced the high-glucose induced cell apoptosis of RMCs through regulation of NF-κB, TGF-β and TRPC6.

The leflunomide has been used in treatment of many diseases. The treatment of arthritis was the most common use for leflunomide. It was reported that leflunomide combined with TNF-α inhibitors could effectively improve rheumatoid arthritis [15]. A study also showed leflunomide could be used in treatment of psoriatic arthritis [16]. It was also reported leflunomide can be used in treatment of diabetes. Yu et al. showed leflunomide had renal protective effect and on inflammatory response of STZ induced diabetic rats [10]. Chen et al. demonstrated leflunomide could control hyperglycemia by increasing AKT and S6K1 phosphorylation in diabetic mice [17]. Zhang et al. reported leflunomide could improve renal injury of DN rats through its inhibition of OPN/TGF-β1 mediated extracellular matrix deposition and tubulointerstitial fibrosis [18]. Another recent study also demonstrated that the combined use of leflunomide and prednisone had good efficiency in treatment of refractory nephrotic syndrome [19]. In the present study, we also showed leflunomide could improve the renal function and reduce the renal injury of DN rats, which was in consistent of other researches.

Application of benazepril in treatment of diabetes has been reported in many studies. Xue et al. showed benazepril hydrochloride could improve DN by decreasing ANGPTL-4 expression [11]. Niu et al. found benazepril could affect integrin-linked kinase and smooth muscle α-actin expression in diabetic rat glomerulus [20]. In an early research, it was found benazepril could also slow progression of renal dysfunction in patients with non-diabetic renal disease [21]. Besides, Jin et al. studied combination use of leflunomide and benazepril in STZ-induced DN rats and found leflunomide and benazepril showed synergistic effects [13]. In our research, it was also demonstrated benazepril could improve the renal function and reduce the renal injury of DN rats. Moreover, we demonstrated the effects of leflunomide and benazepril were through regulation of NF-κB, TGF-β and TRPC6, as well as inhibition of cell apoptosis.

High-glucose induced cell apoptosis was reported in many researches. The injury and cell apoptosis caused by high-glucose are also one of the reasons for diabetic dysfunctions. It was reported that high-glucose-induced apoptosis in human retinal pigment epithelial cells were through the regulation of PTEN [22], it is also found high-glucose could induce cell apoptosis in RMCs [23]. In the present study, we showed for the first time that the inhibitory effects against cell apoptosis by benazepril and leflunomide might be one of the molecular mechanisms to explain the protective effects of benazepril and leflunomide against DN injury.

Both leflunomide and benazepril were reported to inhibit the inflammatory factor of TNF-α, which is regulated by NF-κb signaling [24,25]. Studies also found leflunomide could suppress NF-κb in liver injury and leflunomide metabolite could inhibit NF-κb activation [26,27]. Role of NF-κb, TGF-β and TRPC6 in DN has been reported recently in several researches. NF-κb is thought to be activated and overexpressed in diabetes [28]. It was reported in STZ-induced diabetic rats, the STZ-induced insulin-deficient hyperglycemia caused activation of NF-κB [29]. Iskender et al. also showed in diabetic rats level of NF-κb was enhanced [30]. TGF-β is the downstream protein of NF-κB, which is a pro-sclerotic cytokine widely associated with the development of fibrosis in DN [31]. It was also demonstrated TGF-β/Smad3 was activation in diabetic rats and inhibition of it could improve the diabetes [32].

The transient receptor potential cation channel 6 (TRPC6) is a kind of subfamily of nonselective cation channels permeable to Ca^2+^ [33]. In recent studies, it was found TRCP6 played an important role in inflammation and it mainly promoted inflammation process in diseases such as lung vascular permeability and cardiac fibrosis [34,35]. Besides, TRPC6 is also thought to be regulated by NF-κB in neuron damage [36], and has recently been proven to play important roles in development of DN. Li et al. found the activation of TRPC6 in podocytes was involved in high-glucose induced cell injury [37]. Increased TRPC6 expression is also considered to be associated with tubular epithelial cell proliferation and inflammation in DN [38]. In the present study, we demonstrated for the first time that the combination use of leflunomide and benazepril could reduce the levels of NF-κb, TGF-β and TRPC6 in both DN rats and high-glucose induced RMCs. However deeper insights are still need to give a better understanding.

In conclusion, we conducted both *in vivo* and *in vitro* studies to investigate effect of combination use of leflunomide and benazepril on DN and high-glucose induced RCMs. Results showed the combination use of leflunomide and benazepril could improve the renal function and reduce the renal injury of DN rats and could reduce the levels of NF-κb, TGF-β and TRPC6 in both DN rats and high-glucose induced RMCs. This research could provide research basis for application of leflunomide and benazepril in treatment of DN, as well as give some new research target for DN development.
